# Stepwise-Hierarchical Pooled Analysis for Synergistic Interpretation of Meta-analyses Involving Randomized and Observational Studies: Methodology Development

**DOI:** 10.2196/29642

**Published:** 2021-09-02

**Authors:** In-Soo Shin, Chai Hong Rim

**Affiliations:** 1 Graduate School of Education Dongguk University Seoul Republic of Korea; 2 Department of Radiation Oncology Ansan Hospital Korea University Gyeonggido Republic of Korea

**Keywords:** meta-analysis, observational study, randomized study, interpretation, combination, statistics, synergy, methodology, interpretation, hypothesis, validity

## Abstract

**Background:**

The necessity of including observational studies in meta-analyses has been discussed in the literature, but a synergistic analysis method for combining randomized and observational studies has not been reported. Observational studies differ in validity depending on the degree of the confounders’ influence. Combining interpretations may be challenging, especially if the statistical directions are similar but the magnitude of the pooled results are different between randomized and observational studies (the ”gray zone”).

**Objective:**

To overcome these hindrances, in this study, we aim to introduce a logical method for clinical interpretation of randomized and observational studies.

**Methods:**

We designed a stepwise-hierarchical pooled analysis method to analyze both distribution trends and individual pooled results by dividing the included studies into at least three stages (eg, all studies, balanced studies, and randomized studies).

**Results:**

According to the model, the validity of a hypothesis is mostly based on the pooled results of randomized studies (the highest stage). Ascending patterns in which effect size and statistical significance increase gradually with stage strengthen the validity of the hypothesis; in this case, the effect size of the observational studies is lower than that of the true effect (eg, because of the uncontrolled effect of negative confounders). Descending patterns in which decreasing effect size and statistical significance gradually weaken the validity of the hypothesis suggest that the effect size and statistical significance of the observational studies is larger than the true effect (eg, because of researchers’ bias).

**Conclusions:**

We recommend using the stepwise-hierarchical pooled analysis approach for meta-analyses involving randomized and observational studies.

## Introduction

In the literature, the number of meta-analyses that include observational studies has steadily increased in recent decades [1]. Nevertheless, controversy persists regarding the validity and utility of these meta-analyses. Some researchers are reluctant to assess the validity of the pooled results from studies of a heterogeneous nature and with less robust data. It is clear that compared to the pooled results from randomized studies, the results derived from observational studies may be less representative. Substantial publication of low-quality meta-analyses or those that investigate duplicated topics, which may be empowered by recruiting observational studies, has also been disparaged [2,3].

The abovementioned criticism raises the fundamental question of whether meta-analyses including observational studies should exist in the clinical field. It is clear that randomized studies form the basis of clinical research and have the greatest influence on therapeutic advances and clinical decisions. However, not all decisions in actual clinical practice can be supported only by robust evidence obtained from randomized studies [4]. In particular, it is inevitable that clinical decisions will be made based on observational studies in fields where patients with rare diseases or intractable status are commonly encountered for which there are few available known standard modalities to apply [5]. From a practical perspective, conducting randomized studies requires abundant support, and this support is not available in all medical disciplines. For example, although vendors are willing to support the design of large randomized studies to develop new drugs, if the application of a certain modality has less benefit for the vendor, the driving force for designing a high-quality study will be low.

As an example, in the treatment of liver cancer [6], there is a drug that has demonstrated mild survival gain with little local effect (ie, sorafenib: response rate of ~3%) in the treatment of inoperable cases [7,8]. This drug was studied in phase 3 randomized trials that only proved the survival benefit of the drug for unresectable liver cancers. Although radiotherapy has a significant local effect, with a response rate of over 50%, no phase 3 randomized study has demonstrated a survival gain [9]. Despite this, in a surveillance study on 161 liver cancer clinicians, 86% of physicians stated that they would apply radiotherapy for unresectable liver cancer with major vascular involvement, compared to 66% who would prescribe sorafenib [10]. How were these clinical decisions reached? Clinicians in practice inevitably rely on case series or small observational studies, especially when facing intractable situations in which randomized studies alone cannot support all clinical decisions. In other words, clinicians must perform a self–meta-analysis in their own way involving studies with various designs, commonly including observational studies. The justification for performing meta-analyses that include observational studies can be demonstrated by the necessity to optimize such self–meta-analyses.

In this study, we identify points that require improvement during the process of planning and conducting meta-analyses, and we suggest a method to synergistically interpret results from both nonrandomized and randomized studies.

## Methods

### Identifying Limitations to Overcome

Meta-analyses are performed to aid clinical decision-making in intractable oncologic situations in which a single standard modality has not been established. These meta-analyses must inevitably include observational and randomized studies. The limitations that we recognized must be overcome are as follows.

### Confounders in Observational Studies

When comparing intervention and control groups, the randomization of participants has the advantage of evenly distributing both known and unpredictable confounders [11]. Because of these advantages, randomized studies can allow robust conclusions to be drawn with respect to determining clinical decisions. The main limitation of observational studies is the difficulty of controlling for these confounders. Furthermore, no established method has been presented to quantitatively and objectively measure how such confounders affect pooled estimates. The risk of bias is difficult to control in advance owing to the lack of availability of a protocol [12]. Therefore, the validity of the results is relatively low compared to that of randomized studies. The *Cochrane Handbook for Systematic Reviews of Interventions* states that only observational studies with at least moderate or low risk of bias should be selected in systematic reviews [12]. The Grading of Recommendations, Assessment, Development and Evaluation (GRADE) handbook explains the limitations of observational studies across several categories; it addresses structural issues such as flawed measurement of outcomes and exposure, short follow-up, and inappropriate eligibility criteria, along with inadequate control of confounding. If such a risk exists, it is recommended to downgrade the quality of the study by 1-2 grades according to the degree [13].

On the other hand, as methods of disease assessment advance, more factors are being identified that influence a patient’s prognosis. Recent well-designed observational studies were designed to control a variable number of confounders in the study. A study in which clinical confounders were controlled using methods such as propensity matching and multiple regression analysis [14,15] in a sufficient number of patients should not be analyzed at the same level as studies in which such methods were not used.

In addition, when analyzing observational studies, consideration should be given to how the treatment decisions have been established. Consider two studies that verified the effectiveness of adjuvant radiotherapy after biliary tract cancer surgery (adjuvant radiotherapy for biliary tract cancer has the effect of reducing recurrence, but an increase in survival has not been sufficiently shown) [16]. All related data were obtained from nonrandomized studies. In the first study, the institution decided at a multidisciplinary meeting whether to apply adjuvant radiotherapy; the results were based on comparative data from a single center (adjuvant radiotherapy arm vs no radiotherapy arm). On the other hand, the second observational study compared the results obtained at two independent institutions. The first institution actively performed radiotherapy to maximize treatment efficiency and hospital profits. The second institution did not have a radiation oncology department; therefore, it was necessary to make radiotherapy referrals externally. This situation made surgeons reluctant to recommend radiotherapy, and the patients rarely received adjuvant radiotherapy. In the first study, patients who received radiotherapy were more likely to have prognostic factors related to recurrence. In the second study, the clinical profiles of patients were likely to be evenly distributed between the intervention and control groups.

In summary, observational studies are more likely to be affected by confounders than randomized studies. However, observational studies need to be weighted differently according to their design as well as the degree of control for confounders.

### The “Gray Zone”: Necessity of Combining Interpretations of Randomized and Observational Studies

If there are enough well-designed randomized studies on a subject to be analyzed, there is little need for a meta-analysis including nonrandomized studies. However, the more detailed the clinical topic to be studied and the more incurable the disease, the more difficult it is to make a therapeutic decision using only data from randomized studies. In their randomized sampling analysis of Cochrane reviews, Shrier et al [11] reported that 6 of 16 reviews included 0, 1, or 2 randomized trials. Furthermore, 158 of 183 analyses in 7 additional studies included 2 or fewer randomized studies [11,17]. The reason that randomized studies can control for known and unknown confounders is based on the assumption that the number of participants included in the study is infinite [18]. However, in practice, many randomized studies have difficulty recruiting a sufficient number of patients. Additionally, a blinding process is needed to control for the placebo effect and optimize the design of randomized studies [11]. However, this design is not possible in randomized studies comparing different types of treatment (eg, comparing the effectiveness of lobectomy and radiosurgery in early lung cancer). Limitations such as these necessitate the identification of clinical reasoning, complemented by meta-analyses involving observational studies [19,20].

**Figure 1:**
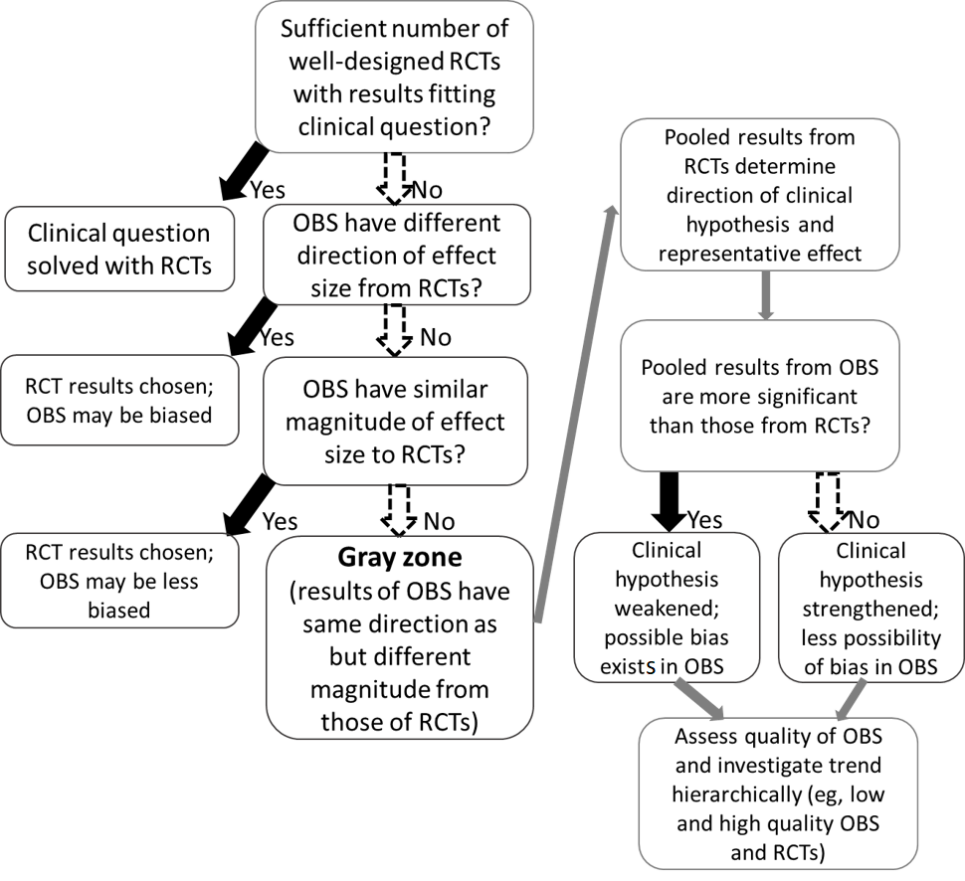
Process by which the “gray zone” is explored and clinical logic flow in the gray zone. OBS: observational studies; RCTs: randomized controlled trials.

In a meta-analysis that includes both randomized and observational studies, if the pooled results of randomized studies and observational studies have similar effect sizes in the same direction without a notable difference in statistical significance, there will be little disagreement in the interpretation of these results. In contrast, if the directions of the two results clearly contradict each other, the majority of scholars will agree to adopt the results of the randomized studies and reject the results of the observational studies, under the assumption that the randomized studies lack significant design flaws. However, there is a “gray zone” where the results of studies with different designs (randomized vs observational studies) have the same direction, but the magnitude of the effect size differs (Figure 1); no clear standard method has been established for combining and interpreting such results. In these situations, the role of pooled results from observational studies may be rather auxiliary if a sufficient number of randomized studies with sufficient validity are recruited. However, as described above, when treating rare diseases or intractable diseases in the clinical field, information from observational studies is necessary for clinical decisions. In other words, it is necessary to complement clinical reasoning based on pooled results of observational studies when the number of randomized studies and the numbers of patients recruited in said studies are insufficient.

### Clinical Logic Flow in the Gray Zone

Physicians should make clinical decisions by using studies with different designs in gray zone situations. Many clinicians review literature found through subjective searches and tend to rely more on research published by authoritative institutions. However, this unsystematic method should be avoided, and a recommended clinical logic flow of interpretation may be as follows:

The pooled results from RCTs determine the direction of the clinical hypothesis and the representative effect size. In the gray zone, complementation from data synthesized from observational studies may be necessary. If the pooled results from observational studies are more significant than those from RCTs, the clinical hypothesis could be weakened and confounding bias could be present among the observational studies. In other words, the clinical hypothesis seems more meaningful in clinical studies with a possibility of bias and a low evidence grade, but it has less significant results than previously expected in high-grade studies such as randomized studies. If the pooled effect of observational studies is less significant, the clinical hypothesis can be strengthened, and there is less possibility of bias. This suggests that clinical hypotheses are less meaningful in clinical studies with possible bias and a low grade of evidence, but more meaningful results are produced in high-grade studies such as randomized studies. Finally, the quality of observational studies can be assessed, and trends of pooled effects according to study design (high- and low-quality observational studies and randomized studies) can be investigated (Figure 1). This process will typically categorize three or more groups, and hierarchical trends can be used to complement clinical hypotheses.

This clinical logical flow will be set as a model and is introduced in detail below.

### Rationale of Stepwise-Hierarchical Pooled Analysis

Stepwise-hierarchical pooled analysis is a method of interpreting the pooled results of studies categorized according to their design and validity. In general, the studies included in a meta-analysis are analyzed by dividing them into at least three groups, and then the individual results of each group and the changing trends among groups are analyzed. In the first level, all studies are analyzed, and in the second level, balanced studies in which major confounders are controlled for are analyzed. Balanced studies are generally defined as those in which major clinical factors are evenly distributed, either based on the study design or statistical method, with additional consideration of the treatment strategy of affiliated institutions whenever possible (discussed in the previous section). Randomized studies can also be included at this stage in the analysis as balanced studies, especially when the number of nonrandomized and balanced studies is small. The final step is to analyze randomized studies. Randomized studies can be analyzed at one level lower if the design is suboptimal (eg, the main clinical factors are not evenly distributed between the intervention and control groups or the randomization method is not reliable) or the number of included patients is too small.

Briefly, the interpretation of stepwise-hierarchical pooled analysis is as follows: The pooled results and statistical significance of the randomized study mainly determine the validity of the hypothesis. When proceeding from an analysis that includes all studies with a low evidence grade to an analysis of more selected studies, this trend further supports the validity of the hypothesis if it is a pattern in which the magnitude and statistical significance of the result increase. However, a decreasing pattern may weaken the validity of the hypothesis, suggesting that there may be biases in the design and results of studies with low evidence ratings.

## Results

### Descriptive Interpretation

**Figure 2:**
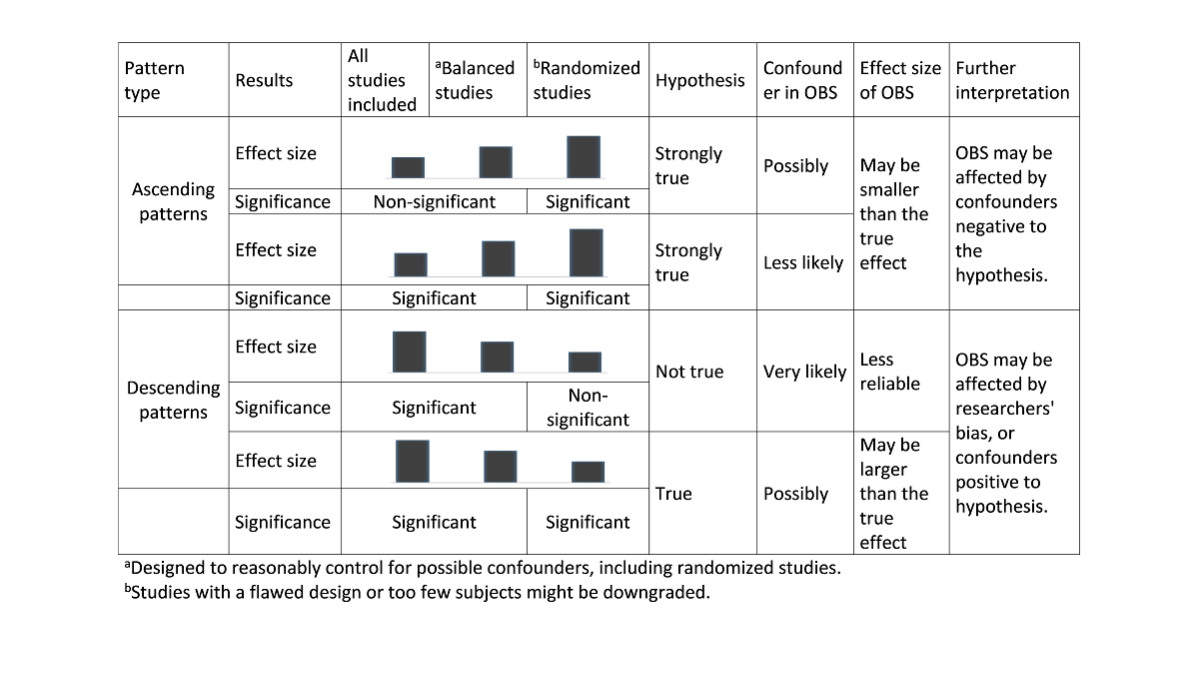
Interpretation of the four representative patterns of stepwise-hierarchical pooled analysis. OBS: observational studies.

The descriptive interpretation of the four representative patterns (Figure 2) is as follows:

1. The effect size and statistical significance increase gradually: The results of the randomized study analysis are statistically significant, and the effect size gradually increases, strengthening the support for the hypothesis. Therefore, the probability is high that the hypothesis is true and strongly positive. The effect size in the observational studies will be lower than the true effect, and if confounders are controlled for, the effect size can be increased. The results of the pooled analyses of observational studies with confounders may not be statistically significant.
2. The effect size gradually increases and the results are statistically significant at all stages: The results of the randomized study analysis are statistically significant, and the pattern of increasing effect size gradually strengthens the reliability of the hypothesis. Therefore, the probability is high that the hypothesis is true and strongly positive. The effect size of observational studies is lower than that of the true effect. Confounders may have a negative effect on the results of observational studies, but because they show statistically significant results, this effect is assumed to be smaller than that in pattern 1.
3. The effect size and statistical significance decrease gradually: The target hypothesis is rejected because the results of the randomized study analysis are not statistically significant. The effect size and statistical significance of the observational studies are not trustworthy. Observational studies are likely to be affected by confounders and researchers’ bias.
4. The effect size gradually decreases and the results are statistically significant at all stages: The target hypothesis is judged to be true because the results of the randomized study analysis are statistically significant. However, the pattern of the effect size gradually decreases, which lowers the reliability of the hypothesis. The effect size of observational studies is larger than the true effect. Observational studies are likely to be affected by confounders and researchers’ bias. Once again, out of the above patterns, the hypothesis is true if the effect sizes are similar in the pooled analyses of both randomized and observational studies, and both analyses are statistically significant. In contrast, if the results of the randomized and observational studies contradict each other, the pooled results of the randomized studies should be weighted more heavily and further investigation of this contradiction should be performed. The stepwise-hierarchical method may not be highly necessary for these situations.

### Examples of Clinical Interpretation

Our team recently published two meta-analyses that used the stepwise-hierarchical method [21,22]. The main results of the two studies showed typical features of the ascending and descending patterns. Therefore, the clinical interpretations of the main results of each paper are discussed as examples.

**Figure 3:**
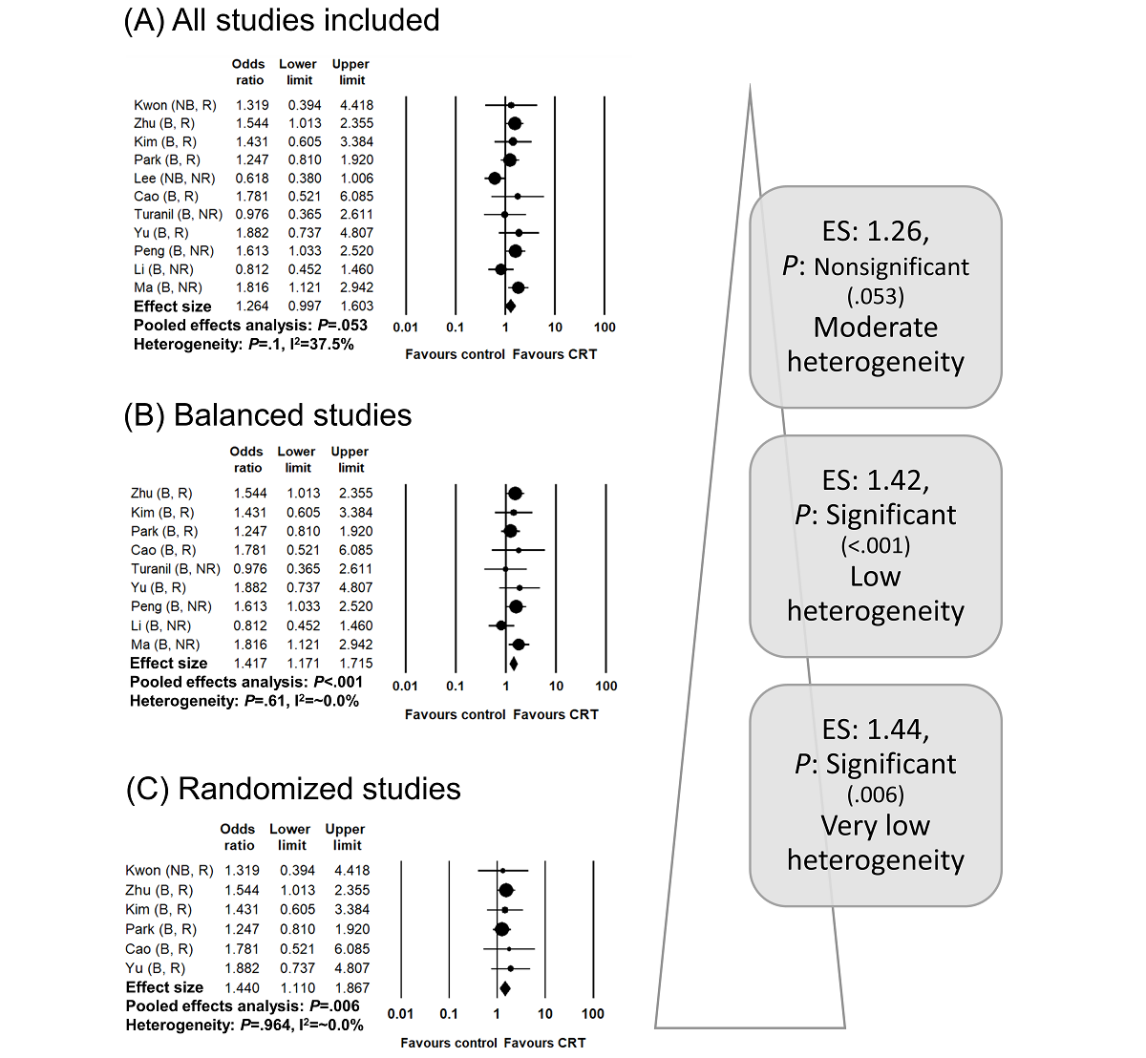
A clinical meta-analysis example of the ascending pattern in the stepwise-hierarchical method based on our previous meta-analysis evaluating the benefits of adjuvant radiochemotherapy after D2 gastrectomy as compared to chemotherapy alone [21]. The forest plots are newly drawn from the raw data obtained by the authors. ES: effect size; CRT: chemoradiotherapy.

The first study focused on adjuvant radiochemotherapy versus chemotherapy after surgery for gastric cancer. In general, the role of additional radiotherapy has not been accepted widely after D2 gastrectomy, including extensive lymphatic dissection [23]. This is because the result for the primary endpoint (disease-free survival) of the only phase 3 randomized study on the subject was marginally nonsignificant [24]. However, several observational studies and small randomized trials have reported the oncologic benefit of radiotherapy [25,26]. Therefore, our team conducted a meta-analysis including randomized and nonrandomized comparative studies to evaluate the disease-free survival benefit of adjuvant radiochemotherapy [21]. As shown in Figure 3, the effect size in the pooled analysis for all studies was 1.264 (95% CI 0.997-1.603), and the *P* value was marginally nonsignificant at *P*=.053. The effect size in the pooled analysis of balanced studies (ie, studies in which major clinical indicators are similarly distributed between arms) was 1.417 (95% CI 1.171-1.715), and the *P* value was highly significant at *P*<.001. The effect size in the pooled analysis of only randomized studies was 1.440 (95% CI 1.110-1.867; *P*=.006), which was also highly significant. The trend of these results correlates with the first of the four typical patterns described above. In other words, the hypothesis of this meta-analysis (radiochemotherapy is significant in reducing disease-free survival after D2 gastrectomy) is strongly supported. The trend in which the effect size increases from considering all studies to considering only balanced or randomized studies strengthened the validity of the hypothesis. The results of observational studies may have underestimated the effect size relative to the true effect due to the influence of confounders (eg, patients assumed to have greater risk of recurrence underwent radiochemotherapy). Furthermore, the low heterogeneity in the analyses of balanced and randomized studies suggests that the pooled results of those studies are reliable and well designed, and they are less affected by possible confounders.

**Figure 4:**
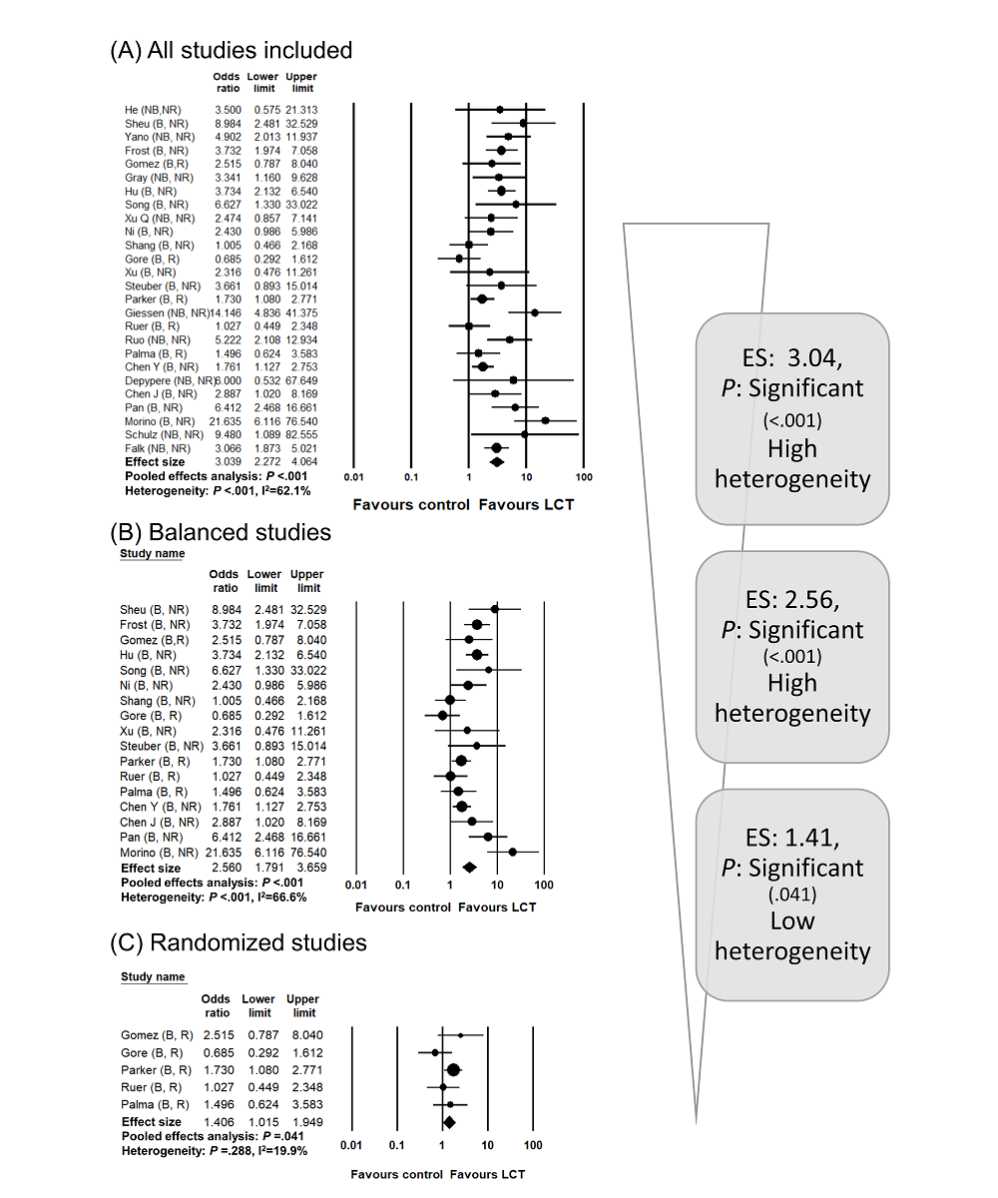
A clinical meta-analysis example of the descending pattern in the stepwise-hierarchical method based on our previous meta-analysis evaluating the benefits of local treatment on oligometastatic disease [22]. The forest plots are newly drawn from the raw data obtained by the authors. ES: effect size; LCT: local consolidative treatment.

The second study was on the benefit of local treatment for oligometastases. Oligometastases refer to a disease state with ≤3 or ≤5 metastatic lesions (definitions differ between studies) [27]. In the recent literature, it was proposed that local treatment for oligometastatic foci could prolong cancer survival [28,29]. Several randomized studies have been published, but the number of patients recruited is generally insufficient [30]. In addition, because the studies in the literature were published according to the type of primary cancer, it was difficult to comprehensively analyze the oncologic benefit of local treatment on general oligometastases. Therefore, we attempted to prove the hypothesis that local treatment for oligometastases will increase overall survival in a meta-analysis [22]. In the analysis of all studies, the pooled effect size was 3.039 (95% CI 2.272-4.064) and the *P* value was significant (*P*<.001). In the analysis of balanced studies, the pooled effect size was 2.560 (95% CI 1.791-3.659), and the *P* value was also highly significant (*P*<.001). In the final analysis of randomized studies, the *P* value was significant (*P*=.04); however, the pooled effect size was 1.406 (95% CI 1.015-1.949), which was smaller in magnitude than that in the previous analyses. The trend of these results correlates with the fourth of the four typical patterns (Figure 4). In other words, the hypothesis of this meta-analysis is true, referring to the analysis results of randomized studies. However, unlike the pattern seen in the meta-analysis of gastric cancer, the change in the effect size or *P* value does not increase the validity of the hypothesis. Observational studies may have been affected by a confounder, and the results may have been larger than the true effect size. Of note, in many studies, local treatment arms had a lower number of metastatic foci than control arms, although the difference was not statistically significant. Unlike the low heterogeneity in the pooled analysis of randomized studies, the high heterogeneity among observational studies suggests the possible effects of confounders. However, such a pattern does not necessarily indicate that the result is weak and not useful. We also found that the benefit of local treatment was higher in certain cancer types (eg, lung cancer, colorectal cancer) and with higher metastatic burden (studies with <5 metastases compared to those with <3 metastases) in further subgroup analyses. Therefore, the authors concluded that although local treatment for oligometastases is beneficial, patients must be carefully selected with consideration of the type of disease or metastatic burden, and the design of future observational studies needs to be improved.

## Discussion

### Principal Considerations

The number of meta-analyses in the literature that include observational studies has been steadily increasing [1]. In actual clinical fields, the decisions that can be fully supported by blinded, randomized studies are limited. It is difficult to assemble a sufficient number of patients free from ethical considerations when the benefits of an intervention are expected to be significant due to observational studies [31]. The treatment methods applied to the intervention and control groups should be of the same type in terms of what the patient perceives. As the understanding of a disease increases and treatment options diversify, it will become increasingly necessary to obtain assistance for therapeutic decisions from meta-analyses of observational studies [17]. Shrier et al [11] described the clinical necessity of meta-analyses including nonrandomized studies; they discussed the practical limitations of randomized studies and explained that well-designed observational studies obtained similar results to those of randomized studies. Vandenbroucke [32] suggested that the reliability of the results can be improved by meta-analyzing observational studies selected in terms of the subject, design, and analysis method.

Other previous publications have discussed the justification for including observational studies in meta-analyses or how to select studies with valid qualities. The *Cochrane Handbook for Systematic Reviews of Interventions* [12], which previously had a conservative perspective on including nonrandomized studies in systematic reviews, added a chapter in the most recent edition on how to assess and interpret these studies in a meta-analysis. Of note, the handbook asserted that only observational studies without a high risk of bias should be included in the meta-analysis. It was also pointed out that there is still no established model that can evaluate how bias or confounders of observational studies affect estimates. However, little is known about how observational and randomized studies should be integrated and analyzed to yield actual clinical decisions.

Limitations of observational studies are categorized and explained in the GRADE handbook [13]. They include fundamental flaws such as inappropriate eligibility criteria, flawed measurement of exposure, inadequate follow-up, and inadequate control of confounders. In the presence of these limitations, it is suggested that the evidence grade should be lowered by one or two steps. Although it is agreeable to evaluate the validity of observational studies in stages, a practical methodology for integrating randomized studies with low- and high-grade observational studies into a formal meta-analysis has not been sufficiently introduced. Indeed, many clinical practice guidelines use GRADE to analyze the grade of evidence and recommendations; those analyses, including observational studies, often rely on narrative reviews. In summary, the necessity to include observational studies in systematic reviews and evaluate their quality has been highlighted in recent literature analytics. However, obtaining clinically useful information by complementing the results of randomized studies with information from observational studies has not been sufficiently suggested.

Recently, the integration of different studies into designs in the field of network meta-analysis has been discussed. In a network meta-analysis, direct and indirect evidence should be analyzed and integrated. A methodology integrating randomized and observational studies has also been studied in the process of synthesizing evidence with different levels of validity [33,34]. Efthimiou et al [35] classified the proposed integrated analysis methods in the literature to date into three categories. These are design-adjusted analyses, in which all trials included in the network meta-analysis involve estimates adjusted according to possible bias and overprecision (based on expert opinions); using informative priors, in which meta-analysis of randomized trials is performed based on priors formulated from meta-analyzing observational studies (Bayesian approach); and three categorical models, in which a meta-analysis is performed for each design, and consequently, the overall effect is acquired by synthesizing all design-specific estimates. Although these approaches have been suggested, according to the scoping review by Zhang et al [36], the vast majority (74%) of network meta-analyses used naïve pooling without specific consideration.

The methods suggested in the field of network meta-analysis and the method of the present study are similar in principle. That is, the results are integrated into a differential consideration of the validity of the evidence. On the other hand, the model of this study is distinct from those suggested in network meta-analysis, in that it is a clinically logical model that analyzes the trend of the synthesized results after differential analysis by considering study quality. In addition, the model proposed in this study is less difficult to apply because it does not require additional statistical analysis or software use. It also has the advantage that clinical interpretation is easy and intuitive, even for physicians without mathematical expertise, because it is based on clinical logical flow. These distinctive features and practical merits provide a summary of the significance of the stepwise hierarchical model, which is a novel method suggested for integration of nonrandomized and randomized studies in frequentist (or classical) meta-analyses.

### Limitations

The limitations of this study are as follows. The four typical patterns described in this study cannot explain all possible patterns and their variations. For a detailed interpretation of clinical decisions, indicators of heterogeneity and publication bias should be interpreted as well. Researchers who are accustomed to making bidirectional decisions based on a specific *P* value of .05 will find the process of analyzing trends in changes in statistical significance unfamiliar [37]. Therefore, quantitative and qualitative interpretation are necessary. Cooperation between a clinician and a biostatistician with sufficient experience in meta-analysis is recommended to successfully use our model. The conclusion empowered by the main results as well as the subgroup results of our second example study can serve as a reference of cooperative interpretation. We expect future meta-analysis studies to use our model and interpret their results, including diverse variations to strengthen the utility of the model and resolve current limitations.

### Conclusions

We recommend using the stepwise-hierarchical pooled analysis approach as a model for interpreting meta-analyses involving randomized and observational studies in a synergistic manner.

## Abbreviations

GRADE: Grading of Recommendations, Assessment, Development and Evaluation
