# Addendum to the Acknowledgments: Stepwise-Hierarchical Pooled Analysis for Synergistic Interpretation of Meta-analyses Involving Randomized and Observational Studies: Methodology Development

**DOI:** 10.2196/33534

**Published:** 2021-09-20

**Authors:** In-Soo Shin, Chai Hong Rim

**Affiliations:** 1 Graduate School of Education, Dongguk University Seoul Republic of Korea; 2 Department of Radiation Oncology Ansan Hospital Korea University Gyeonggido Republic of Korea

In “Stepwise-Hierarchical Pooled Analysis for Synergistic Interpretation of Meta-analyses Involving Randomized and Observational Studies: Methodology Development” (J Med Internet Res 2021;23(9):e29642), two errors were noted.

In the originally published article, the *Acknowledgments* section was incorrect. This section originally read as follows:

This study was supported by the National Research Fund of Korea (NRF-2018R1D1A1B07046998). The funders had no role in the study design, data collection and analysis, decision to publish, or preparation of the manuscript.

This has been corrected to:

This study was supported by the National Research Fund of Korea (NRF-2018R1D1A1B07046998 and NRF-2019M2D2A1A01031560). The funders had no role in the study design, data collection and analysis, decision to publish, or preparation of the manuscript.

As well, in the originally published article, the postal code of the Corresponding Author was incorrectly displayed as "13555." This has been corrected to "15355."

The correction will appear in the online version of the paper on the JMIR Publications website on September 20, 2021, together with the publication of this correction notice. Because this was made after submission to PubMed, PubMed Central, and other full-text repositories, the corrected article has also been resubmitted to those repositories.

